# A 7-Gene Signature Depicts the Biochemical Profile of Early Prefibrotic Myelofibrosis

**DOI:** 10.1371/journal.pone.0161570

**Published:** 2016-08-31

**Authors:** Vibe Skov, Mark Burton, Mads Thomassen, Thomas Stauffer Larsen, Caroline H. Riley, Ann Brinch Madelung, Lasse Kjær, Henrik Bondo, Inger Stamp, Mats Ehinger, Rasmus Dahl-Sørensen, Nana Brochmann, Karsten Nielsen, Jürgen Thiele, Morten K. Jensen, Ole Weis Bjerrum, Torben A. Kruse, Hans Carl Hasselbalch

**Affiliations:** 1 Department of Hematology, Zealand University Hospital, Roskilde, Denmark; 2 Department of Clinical Genetics, Odense University Hospital, Odense, Denmark; 3 Department of Hematology X, Odense University Hospital, Odense, Denmark; 4 Department of Pathology, Naestved Hospital, Naestved, Denmark; 5 Department of Pathology, Lund University Hospital, Lund, Sweden; 6 Department of Pathology, University of Aarhus, Aarhus, Denmark; 7 Institute of Pathology, University of Cologne, Köln, Germany; 8 Department of Hematology L, Rigshospitalet, University of Copenhagen, Copenhagen, Denmark; University of Massachusetts Medical School, UNITED STATES

## Abstract

Recent studies have shown that a large proportion of patients classified as essential thrombocythemia (ET) actually have early primary prefibrotic myelofibrosis (prePMF), which implies an inferior prognosis as compared to patients being diagnosed with so-called genuine or true ET. According to the World Health Organization (WHO) 2008 classification, bone marrow histology is a major component in the distinction between these disease entities. However, the differential diagnosis between them may be challenging and several studies have not been able to distinguish between them. Most lately, it has been argued that simple blood tests, including the leukocyte count and plasma lactate dehydrogenase (LDH) may be useful tools to separate genuine ET from prePMF, the latter disease entity more often being featured by anemia, leukocytosis and elevated LDH. Whole blood gene expression profiling was performed in 17 and 9 patients diagnosed with ET and PMF, respectively. Using elevated LDH obtained at the time of diagnosis as a marker of prePMF, a 7-gene signature was identified which correctly predicted the prePMF group with a sensitivity of 100% and a specificity of 89%. The 7 genes included *MPO*, *CEACAM8*, *CRISP3*, *MS4A3*, *CEACAM6*, *HEMGN*, and *MMP8*, which are genes known to be involved in inflammation, cell adhesion, differentiation and proliferation. Evaluation of bone marrow biopsies and the 7-gene signature showed a concordance rate of 71%, 79%, 62%, and 38%. Our 7-gene signature may be a useful tool to differentiate between genuine ET and prePMF but needs to be validated in a larger cohort of “ET” patients.

## Introduction

The Philadelphia-negative myeloproliferative neoplasms (MPNs) include essential thrombocythemia (ET), polycythemia vera (PV) and primary myelofibrosis (PMF) [1]. Recent studies have shown that a large proportion of patients classified as ET actually have early primary prefibrotic myelofibrosis (prePMF) [2–14]. Since prognosis and likely in the future also treatment of these entities are different, it is important to distinguish between them [8,11]. According to the WHO 2008 classification, bone marrow histology is a major component in the distinction between ET and prePMF [2–14]. However, the differential diagnosis between these two entities may be challenging and some studies have not been able to distinguish between them [15–16]. Most lately, it has been argued that simple blood tests–the hemoglobin (Hb) concentration, the leukocyte count and plasma lactate dehydrogenase (LDH)—may be useful tools to separate genuine ET from early prePMF, the latter disease entity more often being featured by anemia, leukocytosis and elevated LDH [12]. Genome wide expression profiling data has been used to perform classification in patients with ET, PV and PMF [17–19]. However, to our knowledge, no study has applied a supervised classification model to distinguish between genuine ET and early prePMF. Herein, we report that a simple gene signature–composed of a few genes–in our whole blood gene expression profiling design [20–26] is able to separate true ET from early prePMF.

## Materials and Methods

Seventeen patients with ET were enrolled in the study. All patients were diagnosed according to the World Health Organization (WHO) 2008 criteria [27] and followed in two institutions in Denmark. Most patients were on cytoreductive therapy, which for the large majority included hydroxyurea (HU). The study was performed in accordance with the Helsinki declaration and was approved by the local ethics committee.

Samples were collected in Paxgene tubes (Preanalytix, Hombrechtikon, Switzerland) and stored at room temperature for 24 hours, then at -20°C for minimum one day, and finally transferred to a -80°C freezer. The Paxgene Blood RNA kit (Qiagen, Franklin Lakes, NJ, USA) was applied to isolate total RNA from whole blood. Quantity of RNA was assessed with the NanoDrop spectrophotometer ND-8000 (NanoDrop Technologies), and RNA integrity was determined with the Agilent 2100 Bioanalyzer (Agilent Technologies, Palo Alto, CA). 300 ng of purified RNA was converted to biotin-labeled amplified RNA (aRNA) using the Message-AmpTM III RNA amplification kit (Ambion, Austin, TX). Fragmented aRNA was hybridized to Affymetrix HG-U133 Plus 2.0 microarray chips (Affymetrix, Santa Clara, CA, USA).

Data preprocessing and statistical analysis of microarrays were done in the R statistical software (www.r-project.org). The robust multi-array average expression measure (rma) was used to perform background correction, normalization, and expression index calculation [28]. Only perfect match probes were used for data analysis. Data are available from Gene Expression Omnibus (http://www.ncbi.nlm.nih.gov/geo; accession no. GSE26049).

Gene expression values for all microarray chips were uploaded to DNA-chip Analyzer (dChip) software [29] and filtering of genes with high variation across all samples was performed. To uncover unknown patterns in the filtered data set, an unsupervised method, principal component analysis (PCA) was applied to the filtered gene list.

Plasma lactate dehydrogenase (LDH) and the leukocyte count at the time of diagnosis were used to divide patients into subgroups with different risks for a specific outcome. Because measurement of plasma LDH in all patients was done using different references, the LDH value required dichotomization into either normal range (genuine ET) or elevated range (prePMF), taking into account the assumption that the large majority of patients with genuine ET have normal or only slightly elevated LDH, whereas patients with prePMF exhibit elevated LDH values. Likewise, patients with leukocyte counts below or above 10.0 x 10^9^/L were assigned to the group of patients with genuine ET or prePMF, respectively. The patients in the genuine ET or prePMF groups are defined as having either low or high risk of developing post-ET myelofibrosis or overt PMF, respectively.

Microarray data were used to develop a supervised classification model to predict the outcome of ET patients. Whole blood from patients with ET and PMF and from healthy controls were used for gene expression profiling. Only 1 of 9 patients with PMF received treatment. Comparing gene expression in PMF patients vs. controls, the 12 genes *MPO*, *CEACAM8*, *ELA2*, *DEFA4*, *OLFM4*, *CRISP3*, *MS4A3*, *CEACAM6*, *CTSG*, *AZU1*, *HEMGN*, and *MMP8* are among the top 20 most significantly upregulated genes. None of these 12 genes is significantly deregulated in ET vs. controls. The remaining 8 top 20 genes are significantly deregulated in ET vs. controls and are therefore not considered relevant for classification [19–25]. Genes deregulated in ET may not be predictive of fibrosis which is one of the diagnostic criteria for prePMF and PMF. Therefore, to separate patients with prePMF from patients with genuine ET, these 8 genes are not included in the gene signature.” In our previous paper [25], a distinct 5-gene signature consisting of *AZU1*, *CTSG*, *ELA2*, *OLFM4*, and *DEFA4* identified patients with advanced MPN; however, in the present paper, we sought to identify a gene signature able to distinguish prePMF (PMF in early stage) from genuine ET. Therefore, to find a gene signature with classification performance in prePMF, these 5 genes were omitted from the 12-gene signature resulting in a 7-gene signature including *MPO*, *CEACAM8*, *CRISP3*, *MS4A3*, *CEACAM6*, *HEMGN*, and *MMP8*. Thus, the genes in the 7-gene signature are chosen based on gene expression results from patients with PMF vs. controls.

**Fig 1:**
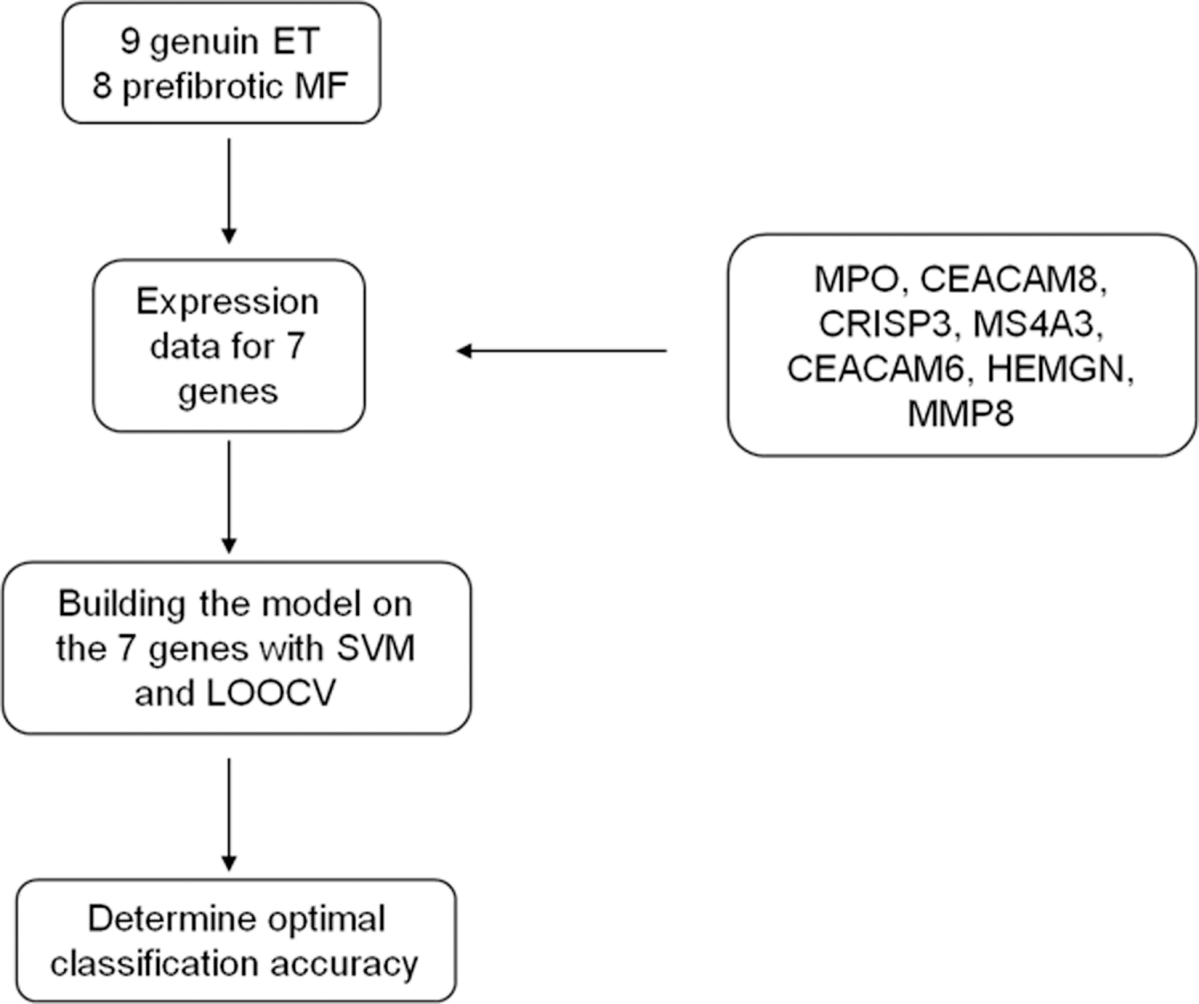
Outline of the classification procedure using SVM and LOOCV. The classification model is built on the 7 genes. The optimal model was selected and classification accuracy determined.

The performance of the 7-gene signature as a classifier in the ET patients was tested using support vector machine (SVM) and leave one out cross validation (LOOCV). Using LOOCV one sample is left out at a time and the rest of the samples is used for classification. Thus, the sample which is left out and classified is not involved in the training process. Gene expression values for the 17 ET patients for the selected 7 genes were uploaded to the SVM classification procedure in the statistical program R using the package *e1071* to build a hyperplane to separate the genes in the data set with maximal margin. Classification and cross validation leaving one sample out at a time (LOOCV) were performed, a probability for prePMF was calculated, and the classification accuracy determined. The model strategy is outlined in Fig 1.

We also tried to perform supervised classification with SVM and LOOCV using the top 500 significant genes obtained from the t-test (genuine ET patients vs. prePMF patients) using both the leukocyte and LDH values obtained at the time of diagnosis to separate the genuine ET patients from the prePMF patients. The performance of the models was assessed from the accuracy values which were quite low, and the models may not be suitable for predicting development of prePMF (data not shown).

Bone marrow biopsies from the time of diagnosis from all 17 patients were evaluated by 4 hematopathologists with extensive experience in the field of MPN classification. Three bone marrow biopsies were not suitable for analysis and therefore excluded from the study. Furthermore, two of the 4 hematopathologists classified one of the ET bone marrow biopsies as unsuitable. Biopsies were fixed in formalin and embedded in paraffin. The sections were stained with hematoxylin and eosin and Gordon and Sweet for reticulin. The biopsies were evaluated strictly according to the WHO-defined morphological criteria [27]. The hematopathologists did not have access to any cytological material and was blinded to all clinical data except patient age. Five diagnoses were used for the histological evaluation: ET, PV, prePMF, overtPMF and “other”.

Literature research and gene ontology analysis were performed to describe the biological function of the 7 genes, and biological network analysis was done using the Igenuity^®^ Pathway Analysis^TM^ Software (IPA, www.ingenuity.com). The IPA software includes the Ingenuity Knowledge Base (IKB) and the Global Molecular Network (GMN) [30]. IKB is a large curated database with a repository of known biological interactions and functional annotations created from peer-reviewed publications of individual studies of genes in human, mouse and rat. The GMN is a large network composed of thousands of genes and gene products that interact with each other. The global network algorithm uses the information from GMN and the user uploaded list of genes to create the optimal networks.

### Ethics statement

The study was approved by The Regional Scientific Ethical Committees for Southern Denmark and was performed in accordance with the Helsinki Declaration. All patients provided written informed consent to participate in the study.

## Results

The clinical parameters for all patients are summarized in Table 1.

**Table 1:** Patient characteristics.

	Number	Age	Gender	JAK2V617F	V617	Therapy	LDH
		(range)	(m/f)	(+/-)	(%)		
Genuine ET	9	62	2/7	5/4	22.1	BU = 1	Normal
		(49–85)			(0.3–67)	ANA = 3	
						HU = 4	
						IFN = 1	
PrePMF	8	65.3	5/3	4/4	21	HU = 6	Elevated
		(35–85)			(0.1–58)	ANA = 2	

Patients are divided into the genuine ET or prePMF group according to the LDH value.

BU = busulphan; ANA = anagrelide; HU = hydroxyurea; IFN = interferon-alpha. LDH = lactate dehydrogenase. Age and V617F are mean and range at the time of blood sampling for gene expression profiling studies. No significant difference in age and V617F % was observed. LDH values at the time of diagnosis.

Based on LDH values obtained at the time of diagnosis, 9 patients were assigned to the genuine ET group and 8 patients were assigned to the prePMF group. According to the LDH value, no difference in age or the *JAK2V617F* % between genuine ET and prePMF was observed. When patients were divided into groups according to the leukocyte value obtained at the time of diagnosis, 9 patients were assigned to the genuine ET group and 8 patients to the prePMF group; however, the composition of the patients in the LDH (Table 1) and leukocyte groups differed (S1 Table)

Of the 54,675 probe sets on the microarray, 1976 genes met the filtering criteria in dChip. Unsupervised PCA applied to the filtered gene list revealed a tendency for the prePMF patients to group together and the genuine ET patients to be more scattered (S1A and S1B Fig). These results suggest that the PCA may not be optimal to separate the two groups of patients.

To explore the performance of a supervised classification model using a predefined gene set, we selected the 7 genes that were among the top 20 most significantly upregulated genes in PMF and not significantly upregulated in ET and not included in the 5-gene signature [25]. The resulting genes included *MPO*, *CEACAM8*, *CRISP3*, *MS4A3*, *CEACAM6*, *HEMGN*, and *MMP8* (S2 Table). In the classification model, the cut off limit for prePMF was set to 0.47 (8/17) which is the number of prePMF patients according to the LDH or leukocyte count divided by the total number of patients.

**Fig 2:**
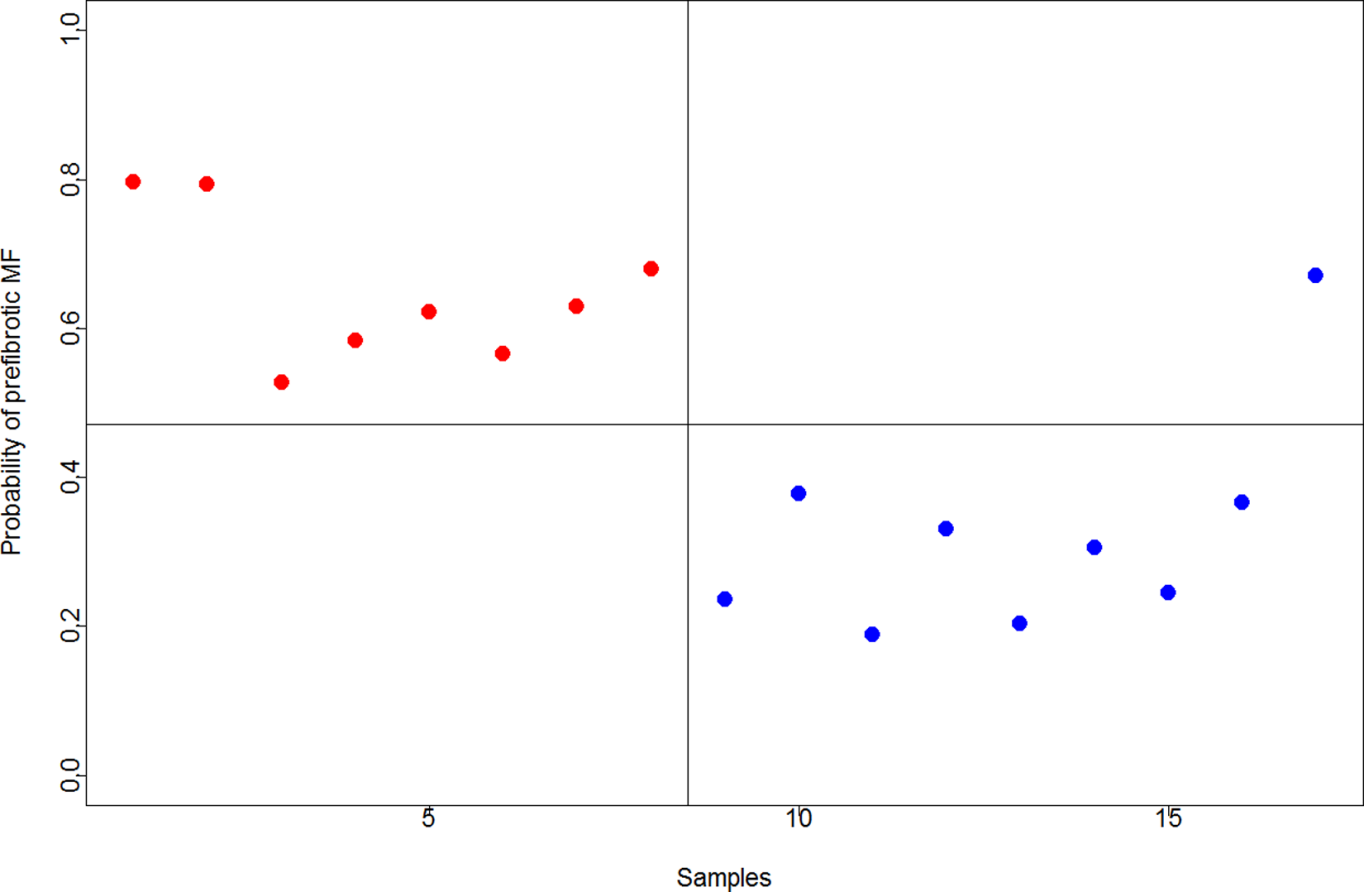
Classification performance of the 7-gene signature in 17 ET patients using LOOCV and SVM. The y-axis shows the probability of developing preMF. The x-axis shows the sample number. Red circles to the left of the vertical line represent patients in the prePMF group and blue circles to the right represent patients in the genuine ET group. Red circles above the horizontal line to the left are correctly predicted patients with prePMF and blue circles below the line to the right are correctly classified as genuine ET patients. Circles below the line to the left and above the line to the right are misclassified patients. Patients are divided according to the LDH value at the time of diagnosis. Balanced accuracy = 94%.

Using LDH as the biochemical variable, the resulting 7-gene signature correctly predicted 8 of 8 patients in the prePMF group (sensitivity 100%) and correctly classified 8 of 9 patients in the genuine ET group (specificity 89%) and showed a balanced accuracy of 94% (Fig 2).

A receiver operating characteristics (ROC) curve with confidence intervals is depicted in S2 Fig. The area under the ROC curve (AUC) is 0.958 showing an excellent separation of genuine ET and prePMF. Using the leukocyte value as the biochemical variable, the 7-gene signature resulted in 62% sensitivity and 78% specificity and showed a balanced accuracy of 70% (data not shown).

The performance of the 7-gene signature using LDH at the time of diagnosis as the biochemical variable was tested against the bone marrow evaluation from the hematopathologists. The concordance between the classification and the 4 hematopathologists was 71%, 79%, 62%, and 38% (Table 2).

**Table 2:** Concordance between classification and bone marrow evaluation.

Classification (LDH)		Bone marrow evaluation											
		Pathologist 1			Pathologist 2			Pathologist 3			Pathologist 4		
		ET	PV	prePMF	ET	PV	prePMF	ET	PV	prePMF	ET	PV	prePMF
ET		6	0	1	6	0	1	3	0	3	1	2	3
preMF		3	0	4	2	0	5	0	2	5	1	2	4

The concordance between the classification and hematopathologist 1, 2, 3, and 4 was 71%, 79%, 62%, and 38%. Three bone marrow biopsies were not suitable for analysis and therefore excluded from the study. Furthermore, pathologist 3 and 4 classified one of the ET bone marrow biopsies as unsuitable. In total, 14 bone marrow biopsies were evaluated by pathologist 1 and 2, and 13 bone marrow biopsies were evaluated by pathologist 3 and 4.

Functional analysis and literature research showed that the 7 genes are involved in key processes associated with the pathophysiology of MPNs (Table 3).

**Table 3:** Annotation of the 7 genes.

Gene Symbol	Gene Title	Functional annotation
MPO	myeloperoxidase	defense response, oxidative stress, inflammation
CEACAM8	carcinoembryonic antigen-related cell adhesion molecule 8	immune response, cell adhesion
CRISP3	cysteine-rich secretory protein 3	immune response, inflammation
MS4A3	membrane-spanning 4-domains, subfamily A, member 3	cell cycle regulator
HEMGN	hemogen	cell differentiation and proliferation
MMP8	matrix metallopeptidase 8 (neutrophil collagenase)	inflammation, cell differentiation and proliferation
CEACAM6	carcinoembryonic antigen-related cell adhesion molecule 6	cell-cell signaling, inflammation, angiogenesis

*CRISP3*, *MMP8* and *MPO* are neutrophil granule proteins playing important roles in immune and inflammatory responses [20, 31–34]. *CEACAM6* and *CEACAM8* are involved in cell adhesion, cellular invasiveness, angiogenesis, and inflammation [20, 35–39]. *MS4A3* is a hematopoietic cell cycle regulator [40], and *HEMGN* plays an important role in hematopoietic development and neoplasms and is also involved in differentiation and proliferation [41–42].

**Fig 3:**
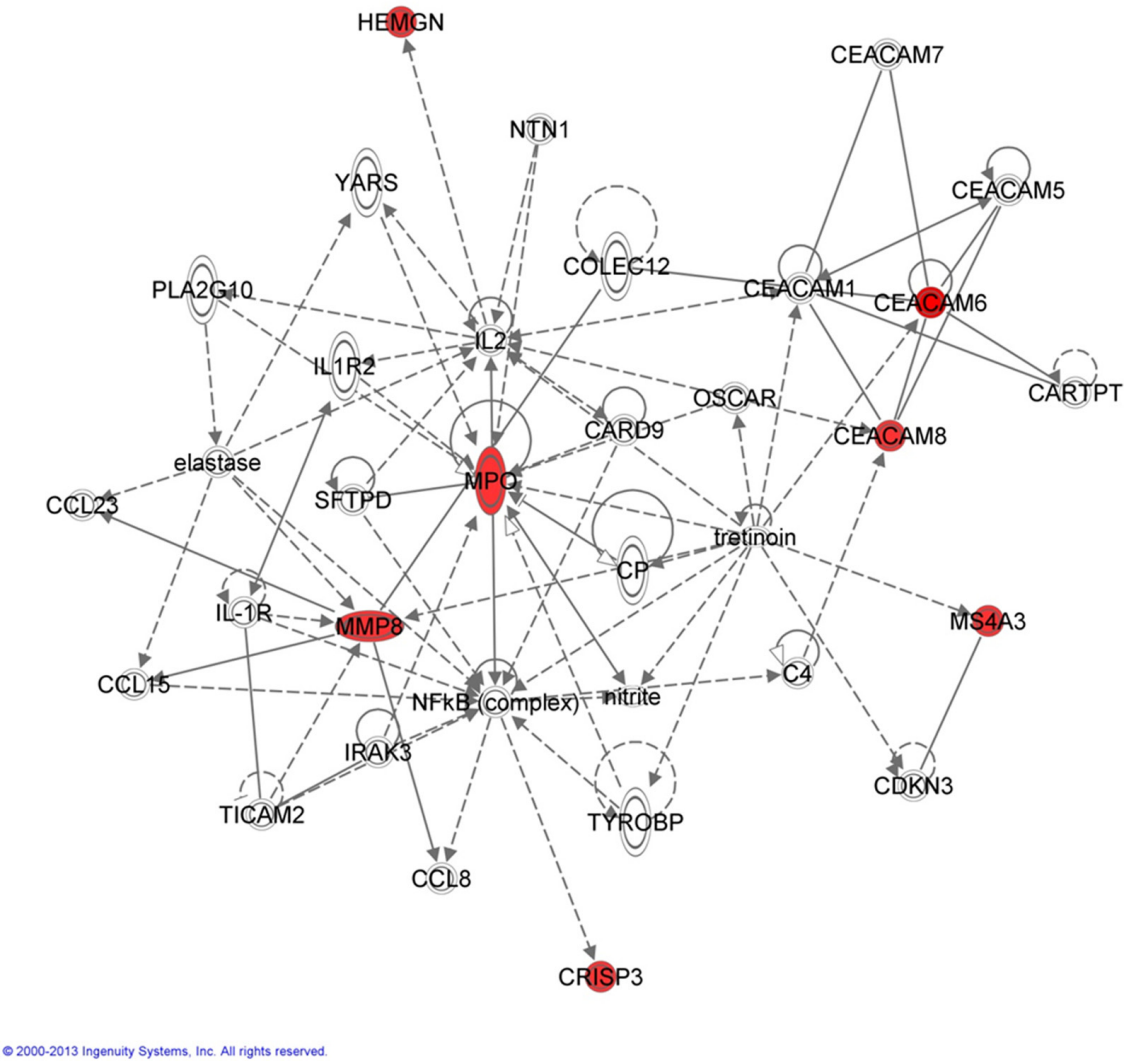
The figure illustrates the network from IPA software analysis. The 7 genes are shown in red. The top functions of the network are inflammatory response, cellular movement and immune cell trafficking. Genes are represented as nodes and the relationship between genes are represented as edges. Solid lines with an arrow: acts on molecule; dashed lines: indirect interaction; solid line: direct interaction.

To identify interactions of the 7 genes with each other and other genes, molecular network analysis was performed using IPA software. The resulting network consists of 34 genes both directly and indirectly connected to the 7 genes (Fig 3).

The top functions of the genes in the resulting network were inflammatory response, cellular movement, and immune cell trafficking well known to be highly relevant in MPNs [20–25]. Furthermore, *NFKB* and *IL2*, which act as hubs—i.e. interacting nodes (genes) with high network degrees assumed to correspond to essential gene products in the cell—in the network, are known to play central roles in inflammation and immune regulation [43–45].

## Discussion

Primary myelofibrosis is aligned along a biological continuum from the early neoplastic stage—prefibrotic myelofibrosis–to the advanced metastatic myelofibrosis stage–“myelofibrosis with myeloid metaplasia” (MMM) [46], which is characterized by bone marrow failure with severe anemia and huge splenomegaly due to extramedullary hematopoiesis with seeding of CD34+ positive cells in the spleen, liver and elsewhere [1,47–48]. The egress of CD34+ cells from the stem cell niches into the circulation has been compared to the metastatic process in solid tumors [49], in which a large number of chemokines, cytokines and proteolytic enzymes are deeply involved [23–26, 50–53]. In this process–as well as in the progression of myelofibrosis from early cancer stage to the advanced cancer stage (MMM)–inflammation and immune deregulation is today considered of major importance [21–23, 53–57]. Thus, the MPNs have been described as “A Human Inflammation Model for Cancer Development” [55], in which chronic inflammation is considered to be the driving force for clonal evolution, increasing genomic instability and additional mutations with ensuing leukemic transformation [53–56]. In recent years, several studies and reviews have supported the concept of chronic inflammation as a highly important factor for disease progression in MPNs as evidenced by a marked increase in several inflammatory cytokines [53]. Furthermore, whole blood transcriptional profiling studies have shown a massive deregulation of inflammation and immune genes and oxidative stress /antioxidative stress genes as well [21–23,26].

The concept “Early Prefibrotic Myelofibrosis” has since 2008 been included in the WHO-classification of MPNs [27]. However, several studies have shown that this disease entity may be difficult to diagnose due to lack of consistency and reproducibility of classical bone marrow features in ET, PV and PMF [6,16] for reasons discussed elsewhere [13]. Thus, bone marrow histology has been criticized for insufficiency in discriminating between the 2 entities and inter-observer variability has been a concern [6]. Regarding genuine ET this entity is histopathologically featured by no or only slight increase in age-matched cellularity, no significant increase in granulo- and erythropoiesis, prominent large to giant mature megakaryocytes with hyperlobulated or deeply folded nuclei, dispersed or loosely clustered in the marrow space and no or very rarely minor increase in reticulin fibers [8]. On the contrary, early prePMF stage is among others characterized by a marked increase in age-matched cellularity with a pronounced proliferation of granulopoiesis and a reduction of erythroid precursors, prominent large to giant atypical megakaryocytes with hyperchromatic nuclei often arranged in dense clusters, and no or no significant increase in reticulin fibers [8]. Clinically, patients with genuine ET have–in general–no enlargement of the spleen, whereas patients with prePMF more often have an enlarged spleen [8,9,11,12]. Importantly, enlargement of the spleen in genuine ET and pr**e**PMF reflects extramedullary hematopoiesis due to egress of CD34+ cells and progenitors from the bone marrow into the circulation to seed preferentially in the spleen and liver. This process is intimately associated with an altered proteolytic micromilieau consequent to in vivo activation of granulocytes and release of their neutrophil granule contents, including e.g. elastase and other proteolytic enzymes [50,51]. This “metastatic “process may not only be driven by clonal activation of neutrophil granule release, elicited by e.g. the *JAK2V617F*-mutation per se [51] but also likely mediated by an inflamed bone marrow milieau [53–56]. Thus, today chronic inflammation is considered to be the driving force behind clonal evolution in MPNs [53–57]. Of note, we have previously shown that patients with PMF exhibit a distinct 5-gene signature composed of several genes of importance for neutrophil granule release (e.g. elastase, defensins) [25]. Furthermore, we have also shown that patients with MPNs are characterized by upregulation of a large number of inflammation and oxidative stress genes [22–23,26] supporting the contention that chronic inflammation may have a major role in MPN pathogenesis [53–57], including the process by which CD34+ cells migrate from the bone marrow to seed in the spleen and liver [22–23,25–26].

When considering some of the previous studies on genuine ET versus early prePMF, it remains a concern that several patients with genuine ET actually had an enlarged spleen and leukocytosis as well [8–9,11]. If strictly adhering to the histopathological description of genuine ET versus early prePMF, patients with genuine ET have no significant granulopoiesis in the bone marrow^8^ and accordingly should have no or no significant leukocytosis and no significant splenomegaly, when taking into account that splenic enlargement implies extramedullary hematopoiesis due to egress of CD34+ cells and progenitors from the bone marrow consequent to enhanced granulopoiesis with in vivo activation of granulocytes and ensuing release of neutrophil granule contents [50–51]. Regardless, a proportion of genuine ET patients in these studies had leukocytosis, significant increase in circulating CD34+ cells and enlarged spleens (the latter accounting for about 15–20%) [8–9,11] but still major findings included significant differences for leukocyte count, platelet count, serum LDH, circulating CD34 cell count, incidence of palpable splenomegaly, and frequency of grade 1 BM fibrosis–all variables being greater in early/prePMF than ET [9].

Our present study adds highly important novel information to the above findings. Firstly, it has unraveled a unique gene signature composed of 7 genes (*MPO*, *CEACAM8*, *CRISP3*, *MS4A3*, *CEACAM6*, *HEMGN*, and *MMP8*), which are capable of distinguishing genuine ET from prePMF with a high sensitivity (100%), specificity (89%) and balanced accuracy (94%) when using LDH at the time of diagnosis as the biochemical variable. By contrast, using the leukocyte value at the time of diagnosis as the biochemical variable, the 7-gene signature resulted in a much lower sensitivity (62%), specificity (78%) and balanced accuracy (70%). Secondly, when testing the performance of the 7-gene signature using LDH at the time of diagnosis as the biochemical variable against the bone marrow evaluation from the hematopathologists, the concordance between the classification and the hematopathologists was 71%, 79%, 62%, and 38%. Thirdly, the 7 genes are involved in key processes associated with the pathophysiology of MPNs, including inflammation and immune deregulation. Thus, *CRISP3*, *MMP8* and *MPO* are neutrophil granule proteins that play very important roles in immune and inflammatory responses [20,31–34]; *CEACAM6* and *CEACAM8* are involved in cell adhesion, cellular invasiveness, angiogenesis, and inflammation [20,35–39] and *MS4A3* is a hematopoietic cell cycle regulator [40]. The *HEMGN* gene plays an important role in hematopoietic development and neoplasms and is also involved in differentiation and proliferation [41–42]. Fourthly, molecular network analysis disclosed 34 genes which both directly and indirectly were connected to the 7 genes. Highly interestingly, the top functions of the genes were inflammatory response, cellular movement, and immune cell trafficking–all considered of utmost importance in the pathogenesis and pathophysiology of MPNs [20–26]. To this end, *NFKB* and *IL2* –in the network acting as hubs i.e. interacting nodes with high network degrees assumed to correspond to essential gene products in the cell, are known to play central roles in inflammation and immune regulation [43–45].

Fifthly, plasma LDH was found to associate much better to our 7-gene signature than the leukocyte count, reflecting plasma LDH to be a more robust biochemical marker of incipient early myelofibrotic transformation than the leukocyte count. The design of our study does not allow us to explore this interesting observation in depth. However, it is intriguing to speculate if elevated plasma LDH levels in prePMF might be indicative of a more enhanced myeloproliferation and turnover of malignant cells than in patients with genuine ET. This interpretation is supported by the inferior survival of early prePMF patients as compared to those patients with genuine ET [9]. It should be noted that a recent study on the value of LDH in distinguishing PMF from the other MPNs displayed serum LDH to be of limited clinical utility [58]. Although serum LDH levels were higher in patients with PMF, levels were also increased in the majority of patients with ET and PV with significant overlap between the subgroups. Serum LDH correlated positively with higher leukocyte and platelet counts, and disease duration in PMF, suggesting LDH to be a biomarker for disease bulk and/or cellular proliferation. It was concluded that LDH lacks specificity for PMF–an observation consistent with the concept of a biological and phenotypic continuum between ET, PV and PMF [22,23,46,58]. In this perspective, our findings of LDH being a better marker than the leukocyte count to associate with our 7-gene signature of inflammation, immune deregulation, proliferation, angiogenesis and cell adhesion in the discrimination between the two entities are only supportive. Thus, elevated LDH may also indicate defective erythropoiesis with concurrent intramedullary and extramedullary hemolysis which are prone to occur in PMF-patients [59–67], in particular in the early myelofibrosis stage, where immune deregulation seems to be more prevalent [59,61–62,66]. Furthermore, the earliest stage in this biological continuum–genuine ET—also includes “triple-negative” ET patients with no *JAK2V617F*, *CALR* or *MPL* mutations and an exceedingly favourable prognosis, suggesting that these patients may have a less severe form of ET or, alternatively, that some of these “ET” patients may actually not have any overt MPN but rather thrombocytosis associated with a more benign process [68–69]. By contrast, patients with early prePMF are already along the road towards “myelofibrosis with myeloid metaplasia” as assessed by more patients with anemia, leukocytosis, elevated LDH and palpable spleens [8–9,11–12,46].

Our study has limitations and strengths. Firstly, it should be taken into account that our patients were not newly diagnosed but investigated at different time points after diagnosis. However, both LDH and leukocyte count were obtained at the time of diagnosis. Secondly, many patients were treated with HU at the time of microarray analysis, which potentially might have impacted the ‘‘global gene signature”. However, in our previous whole blood gene expression studies of MPN-patients on HU treatment, several genes were significantly and increasingly deregulated from the early MPN stage (ET/PV) to the advanced myelofibrosis stage, indicating that HU treatment does not seem to have a major impact upon the different genes being investigated (e.g. inflammation and immune genes) [22–23,26]. Moreover, the 7-gene signature is developed on genes highly significantly deregulated in patients with PMF vs. control subjects with only 1 of 9 PMF patients receiving treatment. Thirdly, we used whole blood instead of e.g. isolated granulocytes, CD34+ cells and mononuclear cells, which have been used in many previous transcriptional profiling studies in MPNs. In the context of exploring distinct genes of value in the discrimination of genuine ET versus prePMF, we believe that such a discriminatory gene expression profile may be more reliably obtained from transcriptional profiling of whole blood than if isolated cell types were analysed. Indeed, our previous studies of whole blood transcriptional profiling are strongly supportive, since these studies–in addition to confirming gene signatures obtained by analyzing single cells by others but generally with much stronger signals–also have unravelled deregulation of several genes which may be involved in disease pathogenesis and progression [20,21–26]. As being underscored elsewhere [26], our approach certainly also makes sense when considering that the ‘‘tumor tissue” being studied–whole blood–is composed of clonal ‘‘tumor” cells (being both myeloid cells, platelets, B-cells and T-cells) and non-clonal cells, including immune cells, which are all activated due to e.g. chronic inflammation and oxidative stress which are considered of crucial importance in MPN-disease pathogenesis [53–57]. In this regard, whole blood transcriptional profiling is actually very similar to all other studies of gene signatures in tumor tissue. Accordingly, in the context of studying powerful gene signatures in the discrimination between genuine ET and early prePMF, our approach with whole blood transcriptional profiling is more a strength than a limitation. Fourthly, our study included a limited number of ET patients. However, the fact that we were able to isolate a 7-gene signature using sophisticated and highly informative gene methodologies in such a small cohort of patients is supportive of our gene signature possessing a high discriminatory power. However, this observation needs to be demonstrated and validated in larger cohorts of ET-patients using our whole blood gene expression profiling design.

In conclusion, we have for the first time shown that a simple 7-gene signature—composed of genes involved in inflammation, immune regulation, cell proliferation, angiogenesis and cell adhesion—is able to discriminate between genuine ET and early prePMF. Considering the concern in regard to reproducibility of histopathological findings to discriminate between these disease entities [6], further studies are urgently needed which by other methodologies aim at identifying and distinctly separating these subsets of MPN-patients. In this regard, a recent study by Pahl et al is of particular importance, showing quantitative *NF-E2* immunohistochemistry of bone marrow biopsies of MPN patients with thrombocytosis to be of value in distinguishing ET from early prePMF [70]. Studies are in progress to validate our results in larger cohorts of “ET” patients with concomitant *NF-E2* immunohistochemistry on bone marrow biopsies. Hopefully, this combinatorial approach using whole blood gene expression profiling together with bone marrow *NF-E2* immunohistochemistry may yield novel important information to be included in decision making on treatment and prognosis of patients with ET and prePMF.

## Supporting Information

S1 Fig Principal component analysis on the 17 ET patients. The analysis was performed on the 1976 genes with highest variation across all samples. Blue circles: genuin ET; red circles: pre-MF. (A) Patients are divided according to the LDH value. (B) Patients are divided according to the leukocyte value.
(TIF)

S2 Fig Receiver operator characteristics (ROC) curve. The ROC curve shows the performance of the 7-gene signature. The area under the ROC curve (AUC) is 0.958 showing an excellent separation of genuine ET and prePMF.”
(TIF)

S1 Table Patients are divided into the genuine ET or pre-PMF group according to the leukocyte value. BU = busulphan; ANA = anagrelide; HU = hydroxyurea; IFN = interferon-alpha. Age and V617F are mean and range at the time of blood sampling for gene expression profiling studies. No significant difference in age and V617F % was observed. Leukocyte values at the time of diagnosis.
(DOCX)

S2 Table Expression profiling of the 7 genes in patients with PMF. Fold changes and false discovery rates (FDR) for the 7 genes in whole blood from patients with PMF compared to controls. FC = false discovery rate; FDR = false discovery rate.
(XLSX)
