# Supplementary figures and images for: A 7-Gene Signature Depicts the Biochemical Profile of Early Prefibrotic Myelofibrosis
Source: PLoS One. 2016 Aug 31;11(8):e0161570. doi: 10.1371/journal.pone.0161570 (PMC5007012; doi:10.1371/journal.pone.0161570)

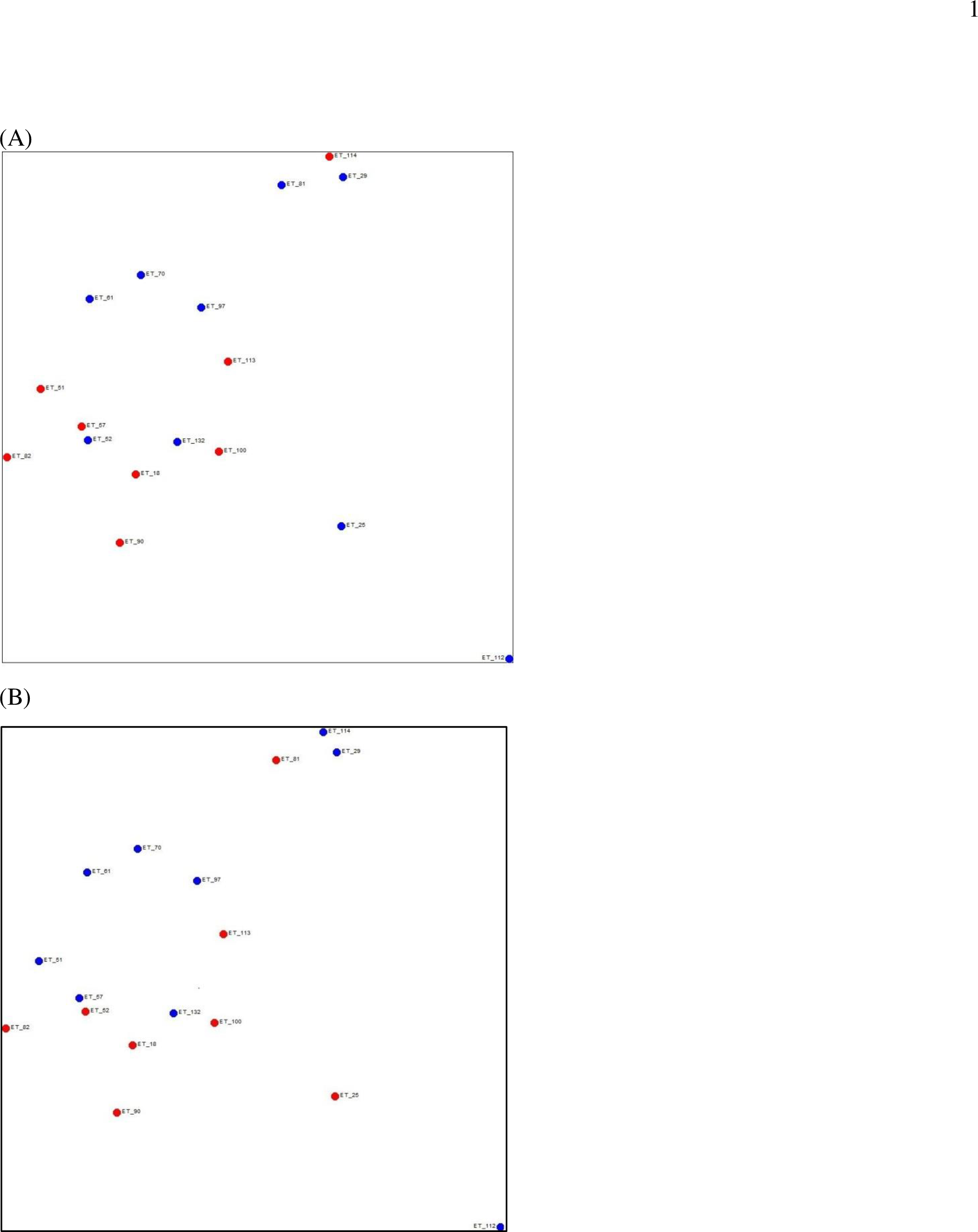

Supplement: S1 Fig — The analysis was performed on the 1976 genes with highest variation across all samples. Blue circles: genuin ET; red circles: pre-MF. (A) Patients are divided according to the LDH value. (B) Patients are divided according to the leukocyte value. (TIF) [file pone.0161570.s001.tif]

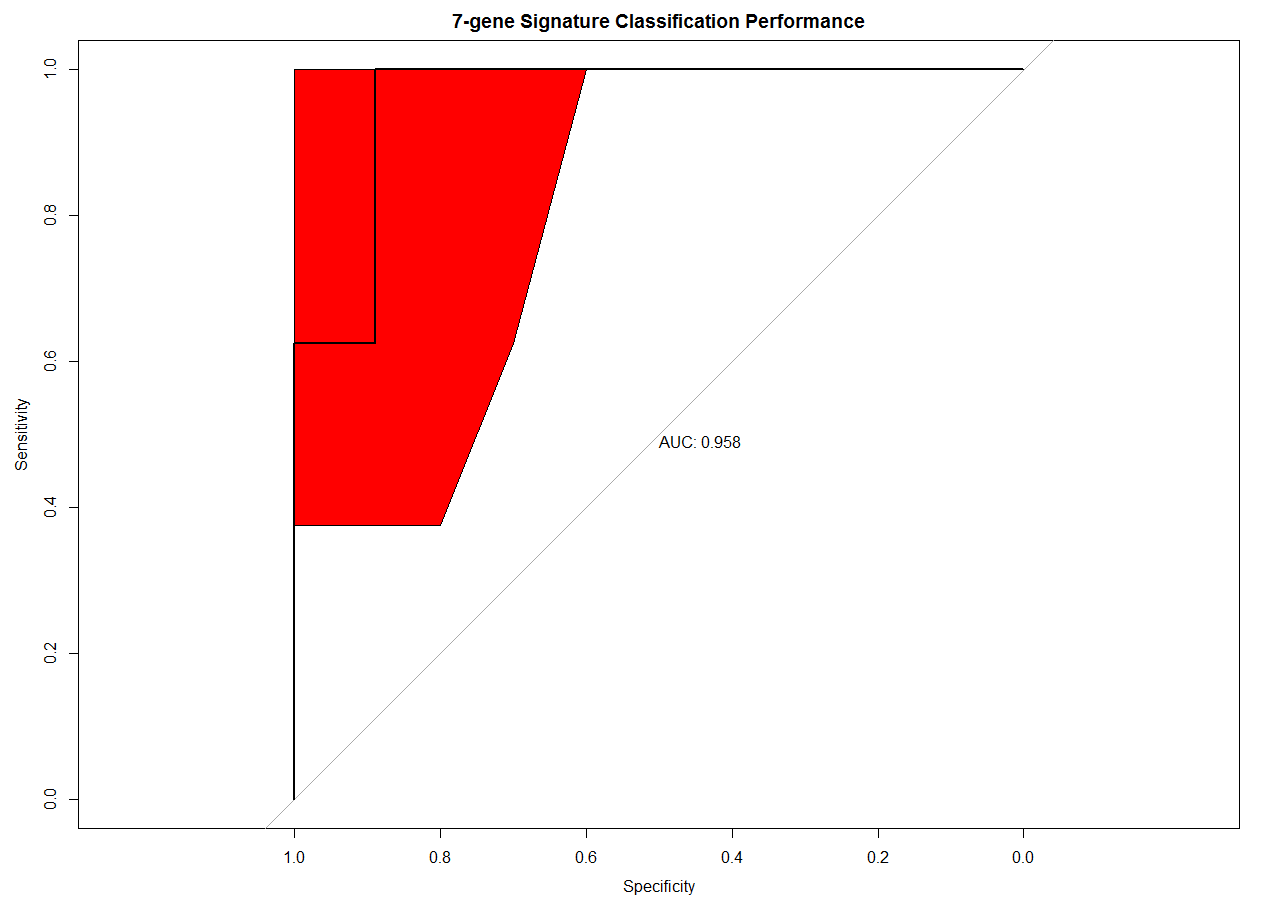

Supplement: S2 Fig — The ROC curve shows the performance of the 7-gene signature. The area under the ROC curve (AUC) is 0.958 showing an excellent separation of genuine ET and prePMF.” (TIF) [file pone.0161570.s002.tif]
