# Supplementary material for: A 7-Gene Signature Depicts the Biochemical Profile of Early Prefibrotic Myelofibrosis
Source: PLoS One. 2016 Aug 31;11(8):e0161570. doi: 10.1371/journal.pone.0161570 (PMC5007012; doi:10.1371/journal.pone.0161570)
Supplement: S1 Table — BU = busulphan; ANA = anagrelide; HU = hydroxyurea; IFN = interferon-alpha. Age and V617F are mean and range at the time of blood sampling for gene expression profiling studies. No significant difference in age and V617F % was observed. Leukocyte values at the time of diagnosis. (DOCX) [file pone.0161570.s003.docx]

|  | Number | Age | Gender  (m/f) | JAK2  V617F  +/- | V617F  % | Therapy | Leukocyte |  |  |  |
| --- | --- | --- | --- | --- | --- | --- | --- | --- | --- | --- |
|  |  |  |  |  |  |  |  |  |  |  |
| Genuine ET | 9 | 60.4  (35-81) | 2/7 | 5/4 | 22  (0.1-67) | BU=1  HU=4  ANA=3  IFN=1 | 7.6  (6.7-9.4) |  |  |  |
| Pre-PMF | 8 | 67  (49-85) | 5/3 | 4/4 | 26.3  (0.3-58) | HU=6  ANA=2 | 12.2  (10.2-14.5) |  |  |  |
